# Interpretability and Class Imbalance in Prediction Models for Pain Volatility in Manage My Pain App Users: Analysis Using Feature Selection and Majority Voting Methods

**DOI:** 10.2196/15601

**Published:** 2019-11-20

**Authors:** Quazi Abidur Rahman, Tahir Janmohamed, Hance Clarke, Paul Ritvo, Jane Heffernan, Joel Katz

**Affiliations:** 1 Department of Computer Science Lakehead University Thunder Bay, ON Canada; 2 Centre for Disease Modelling Department of Mathematics and Statistics York University Toronto, ON Canada; 3 ManagingLife Toronto, ON Canada; 4 Department of Anesthesia and Pain Management Toronto General Hospital Toronto, ON Canada; 5 Department of Psychology York University Toronto, ON Canada; 6 School of Kinesiology & Health Science York University Toronto, ON Canada

**Keywords:** chronic pain, pain volatility, data mining, cluster analysis, machine learning, prediction model, Manage My Pain, pain app

## Abstract

**Background:**

Pain volatility is an important factor in chronic pain experience and adaptation. Previously, we employed machine-learning methods to define and predict pain volatility levels from users of the Manage My Pain app. Reducing the number of features is important to help increase interpretability of such prediction models. Prediction results also need to be consolidated from multiple random subsamples to address the class imbalance issue.

**Objective:**

This study aimed to: (1) increase the interpretability of previously developed pain volatility models by identifying the most important features that distinguish high from low volatility users; and (2) consolidate prediction results from models derived from multiple random subsamples while addressing the class imbalance issue.

**Methods:**

A total of 132 features were extracted from the first month of app use to develop machine learning–based models for predicting pain volatility at the sixth month of app use. Three feature selection methods were applied to identify features that were significantly better predictors than other members of the large features set used for developing the prediction models: (1) Gini impurity criterion; (2) information gain criterion; and (3) Boruta. We then combined the three groups of important features determined by these algorithms to produce the final list of important features. Three machine learning methods were then employed to conduct prediction experiments using the selected important features: (1) logistic regression with ridge estimators; (2) logistic regression with least absolute shrinkage and selection operator; and (3) random forests. Multiple random under-sampling of the majority class was conducted to address class imbalance in the dataset. Subsequently, a majority voting approach was employed to consolidate prediction results from these multiple subsamples. The total number of users included in this study was 879, with a total number of 391,255 pain records.

**Results:**

A threshold of 1.6 was established using clustering methods to differentiate between 2 classes: low volatility (n=694) and high volatility (n=185). The overall prediction accuracy is approximately 70% for both random forests and logistic regression models when using 132 features. Overall, 9 important features were identified using 3 feature selection methods. Of these 9 features, 2 are from the app use category and the other 7 are related to pain statistics. After consolidating models that were developed using random subsamples by majority voting, logistic regression models performed equally well using 132 or 9 features. Random forests performed better than logistic regression methods in predicting the high volatility class. The consolidated accuracy of random forests does not drop significantly (601/879; 68.4% vs 618/879; 70.3%) when only 9 important features are included in the prediction model.

**Conclusions:**

We employed feature selection methods to identify important features in predicting future pain volatility. To address class imbalance, we consolidated models that were developed using multiple random subsamples by majority voting. Reducing the number of features did not result in a significant decrease in the consolidated prediction accuracy.

## Introduction

### Background

Pain is one of the most prevalent health-related concerns and is among the top three most frequent reasons for seeking medical help [1]. Mobile pain apps are transforming how people monitor, manage, and communicate pain-related information [2], and scientific publications on methods and results can help both consumers and health care professionals select the right app to support their treatment plans. Moreover, appropriate analyses can provide valuable insights into pain experiences over long-term periods. We previously conducted two studies [3,4] using data from a pain management app called Manage My Pain [5], where data mining and machine learning methods were employed to understand app usage patterns and define and predict pain volatility. In the first study [3], we divided users into five clusters based on their level of engagement with the app and then applied statistical methods to characterize each user cluster using six different attributes (eg, gender, age, number of pain conditions, number of medications, pain severity, and opioid use).

In the more recent study [4], we developed prediction models to identify and predict groups of users who reported improvements or decrements in their pain levels. To facilitate the development of these models, we addressed the important issue of identifying the most appropriate statistic to use when measuring pain severity over time. We proposed a measure of volatile change that captures fluctuation or variability in pain scores over time. Pain volatility is an important contributor to pain experience for people with chronic pain, particularly because of its linkage with the initiation of opioid addiction [6,7]. Moreover, pain perception and consequent disability are heightened under conditions of greater uncertainty and unpredictability [8], and greater pain volatility is one contributor to uncertainty and unpredictability. Being able to predict future pain volatility can assist patient awareness and application of self-management and appropriate medication use. We defined pain volatility as the mean of absolute changes between 2 consecutive self-reported pain severity ratings (0-10 numeric rating scale). We applied clustering methods to divide users into two classes based on their pain volatility levels: low volatility and high volatility. We then employed four machine learning methods to predict users’ pain volatility level at the sixth month of app use. We developed prediction models where information related to user demographics, pain, medications, and app engagement from the first month’s app use were extracted as features. The total number of features in the prediction models was 130. Prediction models, trained using random forests, performed the best, with the accuracy for the low and the high volatility class reasonably high.

One major drawback of using random forests and similar black-box methods is the lack of interpretability of the trained models. This is especially true when the application domain is medicine and the set of features is large. An interpretable prediction model should incorporate a way to identify a subset of important features that are significantly better predictors than other members of the large features set. This provides health care providers with a practical model to apply and may result in increased confidence in the model. Moreover, the important features may help health care professionals and patients develop appropriate interventions and pain management plans for the future.

While developing volatility prediction models, we addressed the issue of class imbalance because the number of low volatility users was much higher than that of high volatility users. We employed random under-sampling methods to make two equal class sizes. We repeated this under-sampling procedure three times to ensure the stability of the results. Because we did not consolidate the result of these multiple models trained on random subsamples into a single unified model in previous work, we were intent on consolidation in this research.

### Objectives

Accordingly, the present study has two objectives. The first was to identify important features in the pain volatility prediction models that have significantly higher predictive capability than other features. We used two criteria to rank features based on their importance: Gini impurity and information gain. We also applied the Boruta feature selection algorithm to identify a subset of important features. The second objective was to consolidate prediction results from models trained on multiple random subsamples of data where the number of users in the low and high volatility classes was equalized. We employed the majority voting approach to achieve this. Training and testing were conducted using standard 5-fold cross validation. Accuracy for the low and high volatility classes and overall accuracy were calculated to measure and compare the performance of individual and consolidated prediction models developed.

## Methods

### Manage My Pain

Manage My Pain [5], developed by ManagingLife, helps people living with pain to track their pain and functioning daily using an Android or Apple smartphone app. The central feature of Manage My Pain is the pain record that enables users to enter details about their pain experience. Each record contains only one mandatory item, a rating of pain severity using a slider on a visual analogue scale. Users have the option of completing seven more items to describe their pain experience more comprehensively. The app issues daily reminders and prompts users to reflect on their daily accomplishments through a daily reflection*.* Users can also add pain conditions, gender, age, and medications to their profile in the app. As of March 1, 2019, Manage My Pain had 31,700 users and 949,919 pain records.

### Procedure

The present study was reviewed and approved by the Research Ethics Board at York University (Human Participants Review Committee, Certificate number: e2015-160). The user database was accessed and downloaded in two separate files (using plain text format): (1) deidentified user information; and (2) pain records. The user information file contains the following fields: user ID, age at date of app registration, gender, self-reported pain conditions and self-reported medications. The pain record file contains the following fields: user ID, date, pain severity rating (0-10), body location(s) of pain, pain type, pain duration, other associated symptoms, characteristics, relieving factors, ineffective factors, aggravating factors, and environments of pain occurrence. All fields in the text files are delimited using special characters. The data used in this study were downloaded on March 1, 2019. This study covers pain records entered by users between January 1, 2013 and March 1, 2019.

### Data

The primary dataset includes 949,919 pain records from 31,700 users. The outcome period for predicting pain volatility is the sixth month of app usage. The sixth month was chosen because pain lasting at least 6 months meets most generally accepted definitions of chronic pain [9]. In the present study, as in our previous work, we used the first month as the predictor period and we thus collected features from the first month of engagement with Manage My Pain to predict pain volatility during the sixth month of Manage My Pain engagement. The mathematical minimum for calculating pain volatility is 2 pain severity records. However, to increase the reliability of prediction results, users with at least 5 pain records in both the predictor and outcome periods were required for prediction experiments in this study. The number of users in the primary dataset meeting this criterion was 879 and there were 391,255 pain records in the dataset. This is an increase of 97 users and 62,185 records over the number of users and pain records used in our previous study. These 879 users had a mean of 370.09 pain records and a median of 213 pain records.

### Pain Volatility Definition and Prediction

We first briefly summarized the methods used in our previous work [4] to develop volatility prediction models. We defined pain volatility as the mean of absolute changes between two consecutive pain severity ratings within each of the two observation periods. We also applied the k-means clustering method [10] to divide users into two distinct classes (high volatility and low volatility) using a threshold on the pain volatility measure. We extracted 130 features from each user to develop prediction models. Four machine learning methods were employed to develop prediction models: (1) logistic regression with ridge estimators [11]; (2) logistic regression with least absolute shrinkage and selection operator (LASSO) [12]; (3) random forests [13]; and (4) support vector machines (SVM) [14].

The stratified 5-fold cross-validation procedure was used for training and testing. Initial experiments employing 10-fold cross-validation produced similar prediction performance. Data preprocessing was conducted in R (version 3.5.0) (R Core Team, Vienna, Austria). R package glmnet (version 2.0-16) [15] was used for training and testing logistic regression models. We applied the standard Random Forests classification package in WEKA (version 3.8) (University of Waikato, Hamilton, New Zealand) [16], using 100 trees in the Random Forests implementation. The number of features selected at random at each tree-node was set to 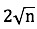, where n is the total number of features. For SVM implementation, we used the WEKA libsvm, employing the Gaussian radial basis function kernel.

The following three measures were used to measure prediction performance:



If we consider users in the low volatility class to be the control group in our experiments, the accuracy of the low volatility class and that of the high volatility class are Specificity and Sensitivity, respectively.

In the present study, the same methods were employed for defining and predicting pain volatility. We added 2 new features to the previous list of 130 features: (1) the standard deviation of the mean of the absolute values of changes between consecutive pain severity ratings; and (2) the absolute value of the difference in pain severity ratings between the end point and the starting point of the trend line of the ratings from the predictor and outcome periods. These two are added to complement 2 existing features: (1) the mean of the absolute values of changes between consecutive pain severity ratings; and (2) the difference in pain severity ratings between the end point and the starting point of the trend line of ratings from the predictor and outcome periods.

Thus, we extracted 132 features from each of the 879 users for developing prediction models. We divided these 132 features into 8 broad categories to facilitate discussions on results from feature selection experiments. The 8 categories are listed below:

1. Demographic (2 features)
  - Gender
  - Age
2. App usage (2 features)
  - Number of pain records (1 features)
  - Number of days with at least one pain record (1 features)
3. Pain statistics (8 features)
  - Mean and standard deviation of pain severity ratings (2 features)
  - Mean and standard deviation of absolute values of changes between consecutive severity ratings (2 features)
  - The difference and the absolute value of the difference between the end point and the starting point of a trend line fitted through the severity ratings (2 features)
  - Pain severity level (1 feature)
  - Pain volatility level (1 feature)
4. Pain descriptors (64 features)
  - Pain locations (24 features)
  - Associated symptoms (20 features)
  - Characteristics (13 features)
  - Environments (7 features)
5. Factors impacting pain (43 features)
  - Aggravating (15 features)
  - Alleviating (14 factors) and Ineffective (14 features)
6. Pain conditions (6 features)
7. Medications (5 features)
8. Mental health conditions (2 features)

### Feature Selection

#### Summary

We applied three different methods to identify features important in predicting pain volatility: the Gini Impurity criterion, the Information Gain criterion, and Boruta.

#### Gini Impurity Criterion

The Gini impurity measure [13] is defined as the probability of an incorrect prediction of a random instance in a dataset, assuming it is randomly predicted according to the distribution of the outcomes in the dataset. This criterion is used while training a Random Forests prediction model [17] to help choose the best feature for splitting a node of a tree. Once a feature has been selected to split a node in a tree in the model, the Gini impurity for the descendants is less than the parent node. The importance is calculated as the difference between the parent node’s impurity and the weighted sum of the children’s nodes’ impurity. This is averaged over the whole forest and a higher mean value indicates higher importance.

#### Information Gain Criterion

For each feature, the information gain [18] measures how much information is gained about the outcome when the value of the feature is obtained. It is calculated as the difference between the unconditional entropy associated with the outcome and the conditional entropy of the outcome given the value of a feature. A higher value of information gain indicates higher importance in prediction.

#### Boruta

Boruta [19] is a wrapper feature selection algorithm built around Random Forests. This method adds randomly permutated copies of all features to the dataset and trains a Random Forests model on this extended dataset. For each feature and its copy, an importance score (mean decreased accuracy) is calculated by the Random Forests training algorithm. A feature is identified to be important by the Boruta algorithm if its importance score is determined to be higher than the best importance score of the permutated copies through a statistical significance test. Similarly, a feature is labeled as not important if the importance is lower than the best importance score of the permutated copies by a statistically significant margin. This process is repeated iteratively until all features have been assigned an important or not important label.

In our prediction experiments, we had five different training sets as we conducted 5-fold cross validation. For each of these five training sets, we applied random under-sampling 5 times, resulting in a total of 25 different training sets. We applied each of the three described algorithms on all 25 sets and identified the features that were common across these sets. We then combined the three groups of important features determined by the three algorithms to produce the final list of important features. 

### Class Imbalance

After defining the low and high volatility classes using the clustering approach, the number of low volatility users is much higher than that of high volatility users (approximately 3 times), as will be detailed in the Results section. In our previous study, we addressed this class imbalance issue by repeated, random under-sampling from the majority class to create a balanced dataset for training prediction models. Under the under-sampling method, instances are chosen at random from the majority class to make the size of the two classes equal. We repeated the under-sampling procedure 3 times to ensure stability of the results.

In the present study, we repeated the under-sampling 5 times and employed a majority voting approach to consolidate the prediction results of these multiple subsamples. In this majority voting method, we assigned a user to the high volatility class when the output was predicted to be high volatility by at least 3 models trained on different subsamples.

## Results

### Prediction Results

**Figure 1:**
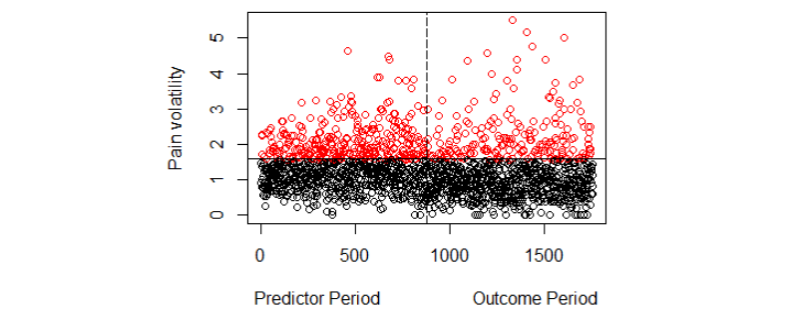
Clustering pain volatility measures. The total number of data points is 1758. Each user has two data points, one each from the predictor and outcome periods. Data points with index (x-axis) 1 to 879 are volatility values from the predictor period and 880 to 1758 are from the outcome period. Black and red colors indicate low and high volatility levels, respectively. The horizontal solid line shows the volatility threshold of 1.6 and the vertical dotted line indicates the cut-off between the predictor and the outcome period.

We first employed the k-means clustering method on the pain volatility measures to distinguish between low and high volatility classes. Figure 1 shows the clustering output. Each of 879 users has two values in the figure: one from the predictor and one from the outcome period. Low and high volatility classes are indicated by black and red colors, respectively. The numerical threshold distinguishing these two classes of users is approximately 1.6, which is the same as our previous study.

We further validated this threshold by reapplying the clustering algorithm on randomly chosen subsamples of the pain volatility values.

**Figure 2:**
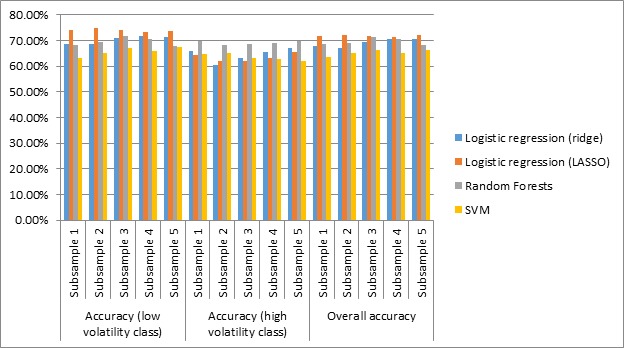
Prediction performance using all 132 features (graphical representation of <xref ref-type="table" rid="table1">Table 1</xref>). LASSO: least absolute shrinkage and selection operator; SVM: support vector machines.

Using the pain volatility threshold of 1.6 resulted in the following division of users in the outcome period: 694 had low volatility and 185 had high volatility. We addressed the class imbalance issue by random under-sampling (repeating 5 times) of the majority class (ie, the low volatility class). We then developed prediction models using logistic regression with ridge estimators and LASSO, random forests, and SVM. The prediction results are shown in Table 1 and Figure 2.

**Table 1:** Prediction performance using all 132 features. Random under-sampling of the majority class (low volatility) was applied and repeated 5 times to make class sizes equal in the training dataset.

Performance measure, subsamples		Logistic regression (ridge), n (%)	Logistic regression (LASSO^a^), n (%)	Random forests, n (%)	SVM^b^, n (%)
**Accuracy (low volatility class; n=694)**					
	Subsample 1	476 (68.6)	513 (73.9)	473 (68.2)	437 (63.0)
	Subsample 2	476 (68.6)	520 (74.9)	482 (69.5)	453 (65.3)
	Subsample 3	492 (70.9)	514 (74.1)	499 (71.9)	465 (67.0)
	Subsample 4	499 (71.9)	509 (73.3)	491 (70.7)	458 (66.0)
	Subsample 5	495 (71.3)	512 (73.8)	471 (67.9)	469 (67.6)
**Accuracy (high volatility class; n=185)**					
	Subsample 1	122 (65.9)	119 (64.3)	129 (69.7)	120 (64.9)
	Subsample 2	112 (60.5)	115 (62.2)	126 (68.1)	118 (65.3)
	Subsample 3	117 (63.2)	115 (62.2)	127 (68.6)	117 (63.2)
	Subsample 4	121 (65.4)	117 (63.2)	128 (69.2)	116 (62.7)
	Subsample 5	124 (67.0)	121 (65.4)	129 (69.7)	115 (62.2)
**Overall accuracy (n=879)**					
	Subsample 1	598 (68.0)	632 (71.9)	602 (68.5)	557 (63.4)
	Subsample 2	588 (66.9)	635 (72.2)	608 (69.2)	571 (65.0)
	Subsample 3	609 (69.3)	629 (71.6)	626 (71.2)	582 (66.21)
	Subsample 4	620 (70.5)	626 (71.2)	619 (70.4)	574 (65.3)
	Subsample 5	619 (70.4)	633 (72.0)	600 (68.3)	584 (66.4)

^a^LASSO: least absolute shrinkage and selection operator.

^b^SVM: support vector machines.

The overall accuracy is approximately 70% for both random forests and logistic regression models. Random forests consistently achieved the same accuracy in predicting both low and high volatility classes across all subsamples. However, SVM did not perform well in predicting future pain volatility levels. Therefore, SVM was not used for features selection experiments in this study.

### Feature Selection Results

**Figure 3:**
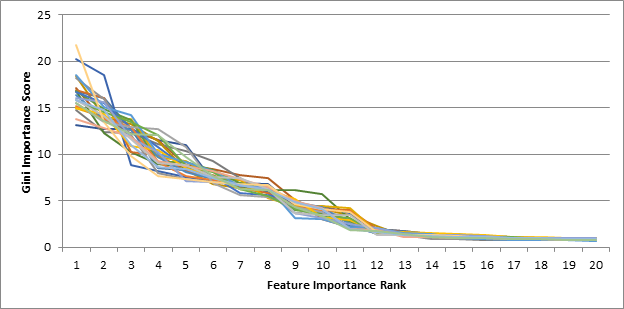
Ranking of importance of features according to Gini importance criterion for all 25 different training sets.

We first used the Gini importance criterion to identify important features in distinguishing high from low volatility users. Mean decreased Gini was calculated for each training set. In 5-fold cross validation experiments, we had 5 different training sets. As we conducted under-sampling 5 times for each training set, we eventually had 25 training sets for this study. For each of these 25 training sets, we trained models using random forests and all 132 features. We then calculated the Gini importance score for each feature to create a ranking based on importance. In Figure 3, we show this importance score for the top 20 features of all 25 training sets.

The importance of features does not decrease significantly beyond the top 11 features in all the training sets. In the list of these top 11 features, we identified 8 that were common across all training sets. They are: (1) the number of days with at least one pain record; (2) the number of pain records; (3) the mean pain severity rating; (4) the standard deviation of the pain severity ratings; (5) the mean of the absolute changes between consecutive pain ratings; (6) the standard deviation of the absolute changes between consecutive pain ratings; (7) the change between the start and end of the trend line fitted through the severity ratings; and (8) the absolute value of the change between the start and end of the trend line fitted through the severity ratings.

**Figure 4:**
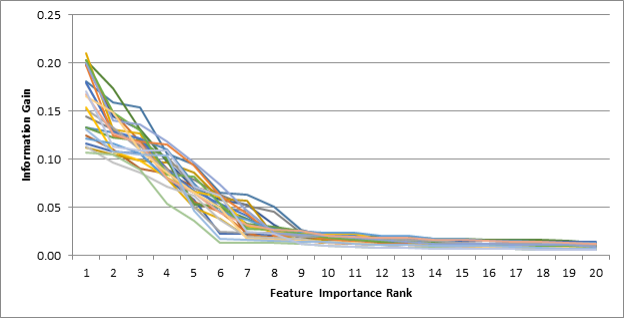
Ranking of features based on the importance calculated using information gain for all 25 different training sets.

The second criterion that we used to calculate the importance of features was information gain. Figure 4 shows the top 20 features in all 25 training sets and the corresponding information gain values.

The information gain drops significantly between the sixth and the ninth feature across different training sets. The following features are the common ones among the top features as ranked by the information gain criterion: (1) the number of days with at least one pain record; (2) the standard deviation of the pain severity ratings; (3) the mean of the absolute changes between consecutive pain ratings (pain volatility scores); (4) the standard deviation of the absolute changes between consecutive pain ratings; and (5) the pain volatility levels in the predictor period.

Lastly, we applied the Boruta method to identify important features in each of the 25 training sets. The number of features considered important by this method varied between 4 and 7 across training sets, with the following 4 common in all sets: (1) the number of days with at least one pain record; (2) the standard deviation of the pain severity ratings; (3) the mean of the absolute changes between consecutive pain ratings; and (4) the standard deviation of the absolute changes between consecutive pain ratings.

Combining the features identified to be important by the three methods leads to the following 9 features: (1) the number of days with at least one pain record; (2) the number of pain records; (3) the mean pain severity rating; (4) the standard deviation of the pain severity ratings; (5) the mean of the absolute changes between consecutive pain ratings (pain volatility scores); (6) the standard deviation of the absolute changes between consecutive pain ratings; (7) the change between the start and end of the trend line fitted through the severity ratings; (8) the absolute value of the change between the start and end of the trend line fitted through the severity ratings; and (9) the pain volatility levels in the predictor period.

The first 2 features are related to app usage and the other 7 features are from the pain statistics category. We used these 9 features to develop volatility prediction models using random forests and logistic regression methods. We then applied majority voting to consolidate the five models developed using subsamples. The prediction results of the individual and consolidated models are presented in Table 2. In Table 2, random under-sampling of the majority class (low volatility) was applied and repeated 5 times to make class sizes equal in the training dataset. Although low volatility and overall accuracy is higher for logistics regression models, random forests perform better when predicting high volatility class across different random subsamples.

**Table 2:** Prediction performance using the 9 selected important features.

Performance measure		Logistic regression (ridge), n (%)	Logistic regression (LASSO^a^), n (%)		Random forests, n (%)
**Accuracy (low volatility class; n=694)**					
	Subsample 1	510 (73.5)		513 (73.9)	461 (67.4)
	Subsample 2	518 (74.6)		516 (74.4)	475 (68.4)
	Subsample 3	511 (73.6)		518 (74.6)	474 (68.3)
	Subsample 4	511 (73.6)		506 (72.9)	454 (65.4)
	Subsample 5	504 (72.6)		506 (72.9)	455 (65.6)
	Consolidated	510 (73.5)		515 (74.2)	476 (68.6)
**Accuracy (high volatility class; n=185)**					
	Subsample 1	114 (61.6)		122 (65.9)	119 (64.3)
	Subsample 2	116 (62.7)		117 (63.2)	129 (69.7)
	Subsample 3	114 (61.6)		115 (62.2)	121 (65.4)
	Subsample 4	118 (63.8)		121 (65.4)	124 (67.0)
	Subsample 5	120 (64.9)		119 (64.3)	123 (66.5)
	Consolidated	115 (62.2)		121 (65.4)	125 (67.6)
**Overall accuracy (n=879)**					
	Subsample 1	624 (71.0)		635 (72.2)	587 (66.8)
	Subsample 2	634 (72.1)		633 (72.0)	604 (68.7)
	Subsample 3	625 (71.1)		633 (72.0)	595 (65.8)
	Subsample 4	629 (71.6)		627 (71.3)	578 (65.8)
	Subsample 5	624 (71.0)		625 (71.1)	578 (65.8)
	Consolidated	625 (71.1)		636 (72.4)	601 (68.4)

^a^LASSO: least absolute shrinkage and selection operator.

**Figure 5:**
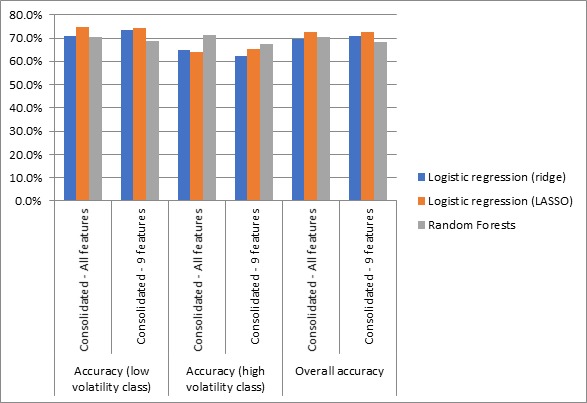
Comparison of consolidated prediction accuracy achieved by all 132 features versus the 9 selected important features. LASSO: least absolute shrinkage and selection operator.

We applied majority voting to the models developed using all 132 features to compare to the consolidated performances of the models developed using the 9 selected features. Figure 5 shows the comparative performance of the consolidated models developed using 132 or 9 features.

Logistic regression–based models perform equally well when developed using 132 or 9 features. This was expected because both methods used regularization to reduce the magnitude of some features’ coefficients. As such, the effect of a redundant feature was significantly diminished even when all features were included in the model.

Random forests performed better than logistic regression methods in predicting the high volatility class. The consolidated overall accuracy measure did not drop significantly (601/879; 68.4% vs 618/879; 70.3%) when only 9 important features were included in the prediction model. The consolidated model’s accuracy was very close to the best performing model (Subsample 2) and was better than four other models (Subsample 1, Subsample 3, Subsample 4, and Subsample 5). Thus, consolidating prediction output from models trained on multiple random subsamples using majority voting performs well when random forests are employed with all features as well as selected important features.

**Figure 7:**
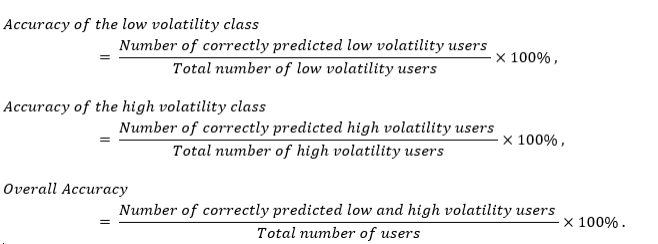
Standalone Equation 2.

## Discussion

### Principal Findings

In this study, we identified features with high predictive capability that could play an important role in predicting pain volatility in users of Manage My Pain, a digital health app for recording pain experiences. Initially, 132 features were extracted, and four methods were used to develop prediction models (logistic regression with ridge estimators, logistic regression with LASSO, random forests, and SVM). We used Gini impurity and information gain criteria to rank features based on their importance. We also employed the Boruta feature selection method to identify a subset of important features. We conducted 5-fold cross validations for training and testing, and repeated random under-sampling 5 times to address the class imbalance issue. Thus, there were 25 different training sets, and for each feature selection method the common important features across all these 25 sets were identified. Finally, we combined the feature sets selected by the three methods to create a list of 9 important features. Two of these 9 features are from the app usage category and the other 7 are from users’ self-reported pain statistics.

Majority voting was utilized to consolidate prediction models trained on the multiple random subsamples to address class imbalance in the dataset. This method worked effectively in achieving the prediction performance closest to the best performing trained model. After feature reduction, the prediction accuracy achieved by two logistic regression methods did not decrease. This shows that the regularization technique embedded in these two methods effectively minimized the impact of redundant features. The consolidated random forests–based models using 9 selected features achieved approximately 68% accuracy for both low and high volatility classes. This is close to the 70% accuracy achieved when models were developed using all 132 features. Logistic regression methods performed better than random forests in predicting the low volatility class while random forests achieved better accuracy for the high volatility class.

### Major Contributions and Future Work

This study continues two prior studies [3,4] where data mining and machine learning methods were used to analyze mobile app users’ pain data. We effectively reduced the set of 132 features drawn from 8 different categories to 9 important features from 2 categories, extracted over the first month of app use, to predict pain volatility at the sixth month. We achieved this without significant reduction in prediction accuracy. Thus, the prediction models developed can be more effectively interpreted and applied as reducing the number of features from 130 to 9 with little loss in accuracy aids interpretability. This is in part because accuracy and facility of medical decision-making depends on the quantity and complexity of information [20]. Health care providers and patients have a limited capacity to absorb and synthesize a predictor set that contains 130 features, making interpretability a challenge. Moreover, increasing interpretability by reducing the important features to a set of 9 may help health care professionals and patients develop appropriate interventions and pain management plans for the future.

Moreover, the approach of majority voting performed well in consolidating models trained on multiple random subsamples while also addressing the class imbalance issue. Notably, random forests using the selected 9 important features performed better in predicting the high volatility class than logistic regression methods. Correctly predicting future high volatility patients is desirable in many ways even with a minor reduction in the accuracy of low volatility prediction. Accordingly, identifying the important features through the 3 different methods used in this study and then developing nonlinear prediction models using random forests is preferable to developing linear models using logistic regression methods.

In our previous study [4] we noted that mean pain intensity among those affected by chronic pain tends not to change significantly over time, given that the pain is, by definition, chronic. As such, mean changes are not always informative, whereas volatility, the degree of change in pain the person must cope with daily, weekly, or hourly, can be helpful in adaptation and management plans. In this current study, 6/9 of the features that were deemed important were useful in measuring change in pain during the predictor period. This variability in pain during the predictor period strongly predicts future volatility at six months. However, two other interesting features related to app use were also significant predictors of pain volatility in our experiments: the number of pain records and the number of days when users created pain records. While the number of pain records may be interpreted as the number of data points in the predictor period, the significance of the number of days and the correlation between these 2 features requires additional analysis. In the future, we shall conduct additional analyses to investigate the possible multicollinearity among the 9 important features and shall also analyze the effects of interactions between features on future pain volatility.

### Limitations

It is usually recommended that datasets used in analytics research are made publicly available for reproducibility and independent verification. However, ManagingLife, the developers of Manage My Pain, is a private organization that serves as the custodian of the data collected by users of Manage My Pain. To ensure it complies with privacy legislation and its own internal privacy policy, ManagingLife cannot make its users’ data public.

## Abbreviations

LASSO: least absolute shrinkage and selection operator
MMP: Manage My Pain
SVM: support vector machines
